# Clinical efficacy and safety of acupuncture versus Western medicine for insomnia: a systematic review and meta-analysis

**DOI:** 10.3389/fneur.2025.1589535

**Published:** 2025-11-14

**Authors:** Jun Ma, Meng Peng, Xue-Jiao Xu

**Affiliations:** Heilongjiang University of Chinese Medicine, Harbin, Heilongjiang, China

**Keywords:** insomnia, acupuncture, Pittsburgh Sleep Quality Index, meta-analysis, systematic review

## Abstract

**Introduction:**

Insomnia is a common sleep disorder that has a significant impact worldwide and seriously affects the quality of life of patients. Benzodiazepines and non-benzodiazepines are the conventional means of treating insomnia disorder in modern medicine. Acupuncture, as a traditional Chinese medicine therapy, is widely used in the treatment of insomnia disorder. A large number of clinical studies have confirmed the significant efficacy of acupuncture in the treatment of insomnia disorder. The aim of this study is to compare the efficacy and safety of acupuncture and sedative-hypnotic medications in the treatment of insomnia disorder through systematic review and Meta-analysis. To elucidate the efficacy and safety of acupuncture alone in the treatment of insomnia disorder.

**Methods:**

A comprehensive computerized literature search was conducted from January 2014 to December 2024 across multiple databases, including VIP Database, Wanfang Data, Cochrane Library, Embase, PubMed, and CNKI to identify studies on acupuncture therapy for patients with insomnia disorder. Two independent researchers performed the data extraction and literature screening processes following standardized protocols. The methodological quality of the included studies was assessed using the Cochrane risk-of-bias tool. Subsequently, a meta-analysis was performed using RevMan 5.4 software, incorporating data from 25 randomized controlled trials (RCTs) that met the predefined inclusion criteria.

**Results:**

The meta-analysis results, based on the Pittsburgh Sleep Quality Index (PSQI) scale scores, revealed that acupuncture regimens (MD: −2.52; 95% CI: −3.10 to −1.94; *p* < 0.00001; I^2^ = 94%; n = 2087) were significantly more effective compared to standalone medication.

**Conclusion:**

Acupuncture has demonstrated significant efficacy in treating insomnia disorder, with preliminary evidence suggesting a potentially favorable safety profile and minimal adverse effects. However, existing studies exhibit inconsistencies in adverse event reporting and are generally limited by small sample sizes and methodological flaws. Therefore, future research should employ more rigorous study designs, expand participant cohorts, and conduct higher-quality investigations to further validate its efficacy and safety, thereby establishing more robust conclusions.

**Systematic review registration:**

https://www.crd.york.ac.uk/PROSPERO/view/, CRD420250653347.

## 1 Introduction

Insomnia disorder is a common sleep disorder that often leads to both psychological and physical abnormalities (1). This disorder not only causes physical fatigue and mental discomfort but also places a significant burden on the socio-economic system (2). Studies have shown that approximately 760 million people worldwide meet the clinical diagnostic criteria for insomnia disorder, and the incidence rate has increased by 37% over the past decade (3). Insomnia disorder affects approximately 10–30% of the adult population worldwide. In Europe and America, the prevalence rate reaches 15–20% (4) and in China, about 35% of the population suffers from acute or chronic insomnia disorder, with an incidence rate of 10 to 20% (5). The clinical manifestation is characterized by persistent difficulty in falling asleep. In mild cases, individuals often struggle to fall asleep, wake up easily after sleeping, and find it hard to fall back asleep. In severe cases, they may suffer from complete sleeplessness throughout the night.

In modern medicine, Western drugs are commonly used to treat insomnia disorder. However, long-term use of these medications may lead to side effects such as addiction, drowsiness, and memory impairment, and their therapeutic efficacy is often limited, making it difficult to effectively control or improve the condition (6). In Traditional Chinese Medicine (TCM), insomnia disorder is attributed to disturbances of heart spirit and imbalances in organ systems. Acupuncture has emerged as an effective therapeutic alternative that integrates traditional meridian theory with evidence-based neurobiological mechanisms. Modern research demonstrates that acupuncture regulates sleep through multiple pathways: by modulating neurotransmitters, reducing inflammatory cytokines, and enhancing neural plasticity via brain-derived neurotrophic factor (BDNF). We specifically focus on acupuncture rather than cognitive behavioral therapy for insomnia disorder (CBT-I) from several considerations. Current systematic evaluations reveal a notable disparity in research development between acupuncture and behavioral therapies. These two therapeutic modalities operate through fundamentally distinct biological pathways. Furthermore, clinical practice observes a growing patient population opting for acupuncture, either as a primary treatment or adjunctive approach, influenced by individual therapeutic preferences and practical constraints in accessing qualified CBT-I providers. In contrast, acupuncture—a widely used traditional Chinese therapy—demonstrates distinct advantages, including simplicity of operation, rapid onset of action, minimal side effects, and significant clinical efficacy. Preliminary evidence also supports its favorable safety profile, making it more acceptable to patients (7). To objectively evaluate the efficacy of acupuncture in improving sleep and alleviating depression, this study conducts a literature quality assessment and meta-analysis of randomized controlled trials on acupuncture and sedative-hypnotic medications for the treatment of insomnia disorder published in the past decade. It compares the effectiveness of sedative-hypnotic medications and acupuncture in treating insomnia disorder, clarifies the efficacy of acupuncture for insomnia disorder, and statistically analyzes the principles of acupoint selection in acupuncture treatment for insomnia disorder. The aim is to provide a scientific reference for the use of acupuncture in treating insomnia disorder.

## 2 Materials and methods

### 2.1 Study registration

The study protocol has been registered with the international prospective systematic review registry PROSPERO (registration number: CRD420250653347). This registration ensures that the study adheres to the intended objectives and technical roadmap and reflects our commitment to transparency and methodological rigor.

### 2.2 Retrieval, methods for research appraisal

To identify RCTs investigating the therapeutic efficacy of acupuncture for insomnia disorder, we conducted a comprehensive systematic literature search across multiple electronic databases. The search spanned from January 2014 to December 2024 and included six major databases: VIP Database, Wanfang Data, Cochrane Library, Embase, PubMed, and CNKI. The search strategy was developed and executed by two independent reviewers with expertise in systematic review methodology. We employed a dual approach to database searching, utilizing both free-text keywords and standardized Medical Subject Headings (MeSH) terms to ensure maximum retrieval sensitivity. The broad search term “acupuncture” was initially adopted to meet the requirements of database retrieval strategies, ensuring the inclusion of various therapies such as manual acupuncture and electroacupuncture while avoiding omissions due to terminology differences or inconsistent indexing. Combined with disease terms like “insomnia” and “sleep disorders” as well as study type terms such as “randomized controlled trial,” this approach aligns with the PICOS framework for literature retrieval and ensures the accuracy of the research scope through subsequent manual screening. The search terms were acupuncture, acupuncture therapy, manual acupuncture, electroacupuncture, insomnia, sleep disorders, difficulty falling asleep, sleep initiation and maintenance disorders, randomized controlled trial and RCT in both Chinese and English. Boolean operators (AND, OR) were strategically employed to optimize search precision. The search strategy was adjusted for each database. To ensure transparency and reproducibility of our search methodology, we have provided a detailed example of our search strategy using PubMed in Table 1. This includes the specific search terms, field tags, and Boolean operators used.

**Table 1:** Search strategy of PubMed.

Number	Search terms
#8	(((“Sleep Initiation and Maintenance Disorders”[Mesh]) OR ((((((((((((((((((((((((((DIMS [Title/Abstract]) OR (Disorders of Initiating[Title/Abstract] AND Maintaining Sleep[Title/Abstract])) OR (Sleeplessness[Title/Abstract])) OR (Insomnia Disorder[Title/Abstract])) OR (Insomnia Disorders[Title/Abstract])) OR (Insomnias[Title/Abstract])) OR (Chronic Insomnia[Title/Abstract])) OR (Insomnia, Chronic[Title/Abstract])) OR (Early Awakening[Title/Abstract])) OR (Awakening, Early[Title/Abstract])) OR (Nonorganic Insomnia[Title/Abstract])) OR (Insomnia, Nonorganic[Title/Abstract])) OR (Primary Insomnia[Title/Abstract])) OR (Insomnia, Primary[Title/Abstract])) OR (Psychophysiological Insomnia[Title/Abstract])) OR (Insomnia, Psychophysiological[Title/Abstract])) OR (Rebound Insomnia[Title/Abstract])) OR (Insomnia, Rebound[Title/Abstract])) OR (Secondary Insomnia[Title/Abstract])) OR (Insomnia, Secondary[Title/Abstract])) OR (Sleep Initiation Dysfunction[Title/Abstract])) OR (Dysfunction, Sleep Initiation[Title/Abstract])) OR (Dysfunctions, Sleep Initiation[Title/Abstract])) OR (Sleep Initiation Dysfunctions[Title/Abstract])) OR (Transient Insomnia[Title/Abstract])) OR (Insomnia, Transient[Title/Abstract]))) AND ((“Acupuncture”[Mesh]) OR (Pharmacopuncture[Title/Abstract]))) AND (randomized controlled trial[Publication Type] OR randomized[Title/Abstract] OR placebo[Title/Abstract])
#7	randomized controlled trial[Publication Type] OR randomized [Title/Abstract] OR placebo[Title/Abstract]
#6	(“Acupuncture”[Mesh]) OR (Pharmacopuncture[Title/Abstract])
#5	Pharmacopuncture[Title/Abstract]
#4	“Acupuncture”[Mesh]
#3	(“Sleep Initiation and Maintenance Disorders”[Mesh]) OR ((((((((((((((((((((((((((DIMS [Title/Abstract]) OR (Disorders of Initiating[Title/Abstract] AND Maintaining Sleep[Title/Abstract])) OR (Sleeplessness[Title/Abstract])) OR (Insomnia Disorder[Title/Abstract])) OR (Insomnia Disorders[Title/Abstract])) OR (Insomnias[Title/Abstract])) OR (Chronic Insomnia[Title/Abstract])) OR (Insomnia, Chronic[Title/Abstract])) OR (Early Awakening[Title/Abstract])) OR (Awakening, Early[Title/Abstract])) OR (Nonorganic Insomnia[Title/Abstract])) OR (Insomnia, Nonorganic[Title/Abstract])) OR (Primary Insomnia[Title/Abstract])) OR (Insomnia, Primary[Title/Abstract])) OR (Psychophysiological Insomnia[Title/Abstract])) OR (Insomnia, Psychophysiological[Title/Abstract])) OR (Rebound Insomnia[Title/Abstract])) OR (Insomnia, Rebound[Title/Abstract])) OR (Secondary Insomnia[Title/Abstract])) OR (Insomnia, Secondary[Title/Abstract])) OR (Sleep Initiation Dysfunction[Title/Abstract])) OR (Dysfunction, Sleep Initiation[Title/Abstract])) OR (Dysfunctions, Sleep Initiation[Title/Abstract])) OR (Sleep Initiation Dysfunctions[Title/Abstract])) OR (Transient Insomnia[Title/Abstract])) OR (Insomnia, Transient[Title/Abstract]))
#2	(((((((((((((((((((((((((DIMS [Title/Abstract]) OR (Disorders of Initiating[Title/Abstract] AND Maintaining Sleep[Title/Abstract])) OR (Sleeplessness[Title/Abstract])) OR (Insomnia Disorder[Title/Abstract])) OR (Insomnia Disorders[Title/Abstract])) OR (Insomnias[Title/Abstract])) OR (Chronic Insomnia[Title/Abstract])) OR (Insomnia, Chronic[Title/Abstract])) OR (Early Awakening[Title/Abstract])) OR (Awakening, Early[Title/Abstract])) OR (Nonorganic Insomnia[Title/Abstract])) OR (Insomnia, Nonorganic[Title/Abstract])) OR (Primary Insomnia[Title/Abstract])) OR (Insomnia, Primary[Title/Abstract])) OR (Psychophysiological Insomnia[Title/Abstract])) OR (Insomnia, Psychophysiological[Title/Abstract])) OR (Rebound Insomnia[Title/Abstract])) OR (Insomnia, Rebound[Title/Abstract])) OR (Secondary Insomnia[Title/Abstract])) OR (Insomnia, Secondary[Title/Abstract])) OR (Sleep Initiation Dysfunction[Title/Abstract])) OR (Dysfunction, Sleep Initiation[Title/Abstract])) OR (Dysfunctions, Sleep Initiation[Title/Abstract])) OR (Sleep Initiation Dysfunctions[Title/Abstract])) OR (Transient Insomnia[Title/Abstract])) OR (Insomnia, Transient[Title/Abstract])
#1	“Sleep Initiation and Maintenance Disorders”[Mesh]

### 2.3 Inclusion and exclusion criteria

Inclusion criteria: (i) Published randomized controlled trials of acupuncture in the treatment of insomnia disorder. The experimental group was treated with acupuncture (the specific acupuncture method and the type of acupoints were not limited), and the control group was treated with sedative-hypnotic medications (e.g., benzodiazepines, non-benzodiazepine hypnotics; the specific type was not limited). (ii) The research objects in the RCT were patients diagnosed with insomnia disorder and those who met the diagnostic criteria of insomnia disorder in traditional Chinese medicine or western medicine. (iii) The outcome indicators were clear, PSQI score, etc. (iv) There are no restrictions on the source, course, gender and age of cases.

Exclusion criteria: (i) Repetitive literature; (ii) Research design type and research Subjects as well as literature on interventions not described in detail; (iii) Comprehensive Narrative literature; (iv) Meta analysis literature there.

### 2.4 Research selection and data extraction

Two independent reviewers were responsible for data extraction and study screening. Initially, they independently screened the titles and abstracts of the retrieved articles. Subsequently, a comprehensive full-text review was conducted for potentially eligible studies. RCTs that met the predefined inclusion criteria were included for qualitative synthesis. For each eligible study, the following data were systematically extracted: study design characteristics (e.g., random sequence generation method), bibliographic information (e.g., author affiliations and publication year), demographic details (e.g., sample size, mean age, and gender distribution), diagnostic criteria used, intervention protocols (including treatment methods for both experimental and control groups), outcome measures, duration of treatment, and other relevant study-specific information. Any discrepancies in data extraction or study selection were resolved through discussion or, when necessary, by consultation with a third reviewer.

### 2.5 Outcomes

The primary outcome measure in this systematic review was sleep quality, as evaluated using the PSQI (8). The PSQI instrument comprises 19 self-reported items that are categorized into seven distinct components, each scored on a scale from 0 to 3. These component scores are subsequently aggregated to produce a global PSQI score ranging from 0 to 21, where higher scores denote poorer sleep quality. Specifically, a global score of 0 represents the absence of sleep difficulties, while a maximum score of 21 indicates severe sleep disturbances across all measured domains.

### 2.6 Statistical, analysis

All statistical analyses were conducted using RevMan 5.4 software. A random-effects model was employed for meta-analysis to account for potential variability across studies. The treatment effect of acupuncture was evaluated using odds ratios (OR) with 95% confidence intervals (CI) for dichotomous outcomes, while standard mean differences (SMD) with 95% CI were calculated for continuous outcomes, including PSQI scores comparing acupuncture and Western medicine interventions. Clinical and methodological heterogeneity among included studies was systematically assessed through longitudinal analyses. Statistical heterogeneity was evaluated using I^2^ statistics, with I^2^ values > 50% indicating substantial heterogeneity. Although low heterogeneity was detected, the random-effects model was maintained due to the recognized limitations of heterogeneity tests, particularly when analyzing a limited number of studies. A threshold of *p* < 0.05 was established for statistical significance. Following this rigorous analytical approach, 25 RCTs met the inclusion criteria and were incorporated into the qualitative synthesis. The methodological framework ensured robust evaluation of both clinical outcomes and study heterogeneity, while maintaining appropriate statistical thresholds for interpretation of results.

## 3 Results

### 3.1 Study selection

**Figure 1:**
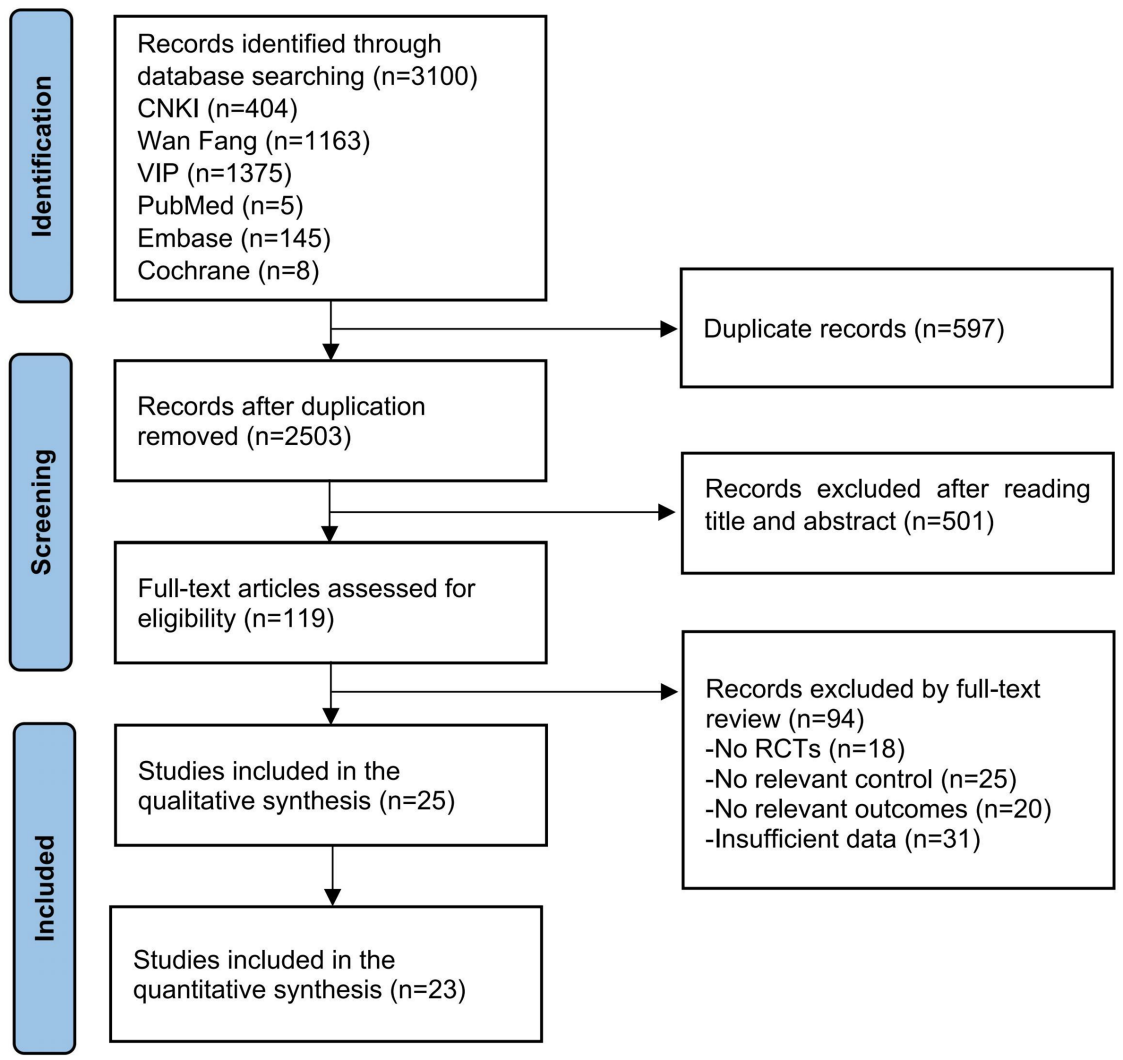
Flow chart of the review process.

The study selection process is illustrated in Figure 1 following the PRISMA guidelines for systematic reviews. Our initial comprehensive database search identified a total of 3,100 potentially relevant publications across multiple electronic databases. Following the removal of 597 duplicate records through EndNote reference management software and manual verification, we proceeded to screen the remaining 2,503 unique records. During the initial screening phase, two independent reviewers systematically evaluated the titles and abstracts against our predefined inclusion criteria, resulting in the exclusion of 2,002 records. This rigorous screening process yielded 501 potentially eligible articles for full-text assessment. In the subsequent phase, the research team conducted a thorough examination of the full texts of these 501 articles. This detailed evaluation process led to the exclusion of 476 publications that failed to meet our inclusion criteria, primarily due to reasons such as inappropriate study design, irrelevant interventions, or inadequate outcome measures. Ultimately, 25 high-quality RCTs were identified as meeting all inclusion criteria and were selected for qualitative synthesis in our systematic review. In these RCT studies, data from weeks 1 through 4 were included in the quantitative synthesis, which comprised a total of 23 studies.

### 3.2 Study characteristics

This systematic review analyzed studies published from 2014 to 2024, encompassing a pooled sample of 2,087 participants across all eligible trials, as systematically presented in Table 2. The included publications were exclusively published in either English or Chinese, demonstrating the international scope of research in this therapeutic domain. All studies implemented rigorously standardized diagnostic protocols, diagnostic criteria of Western medicine include the Diagnostic and Statistical Manual of Mental Disorders, Fifth Edition (DSM-V) (9), the International Classification of Diseases, tenth revision (ICD-10) (10) diagnostic criteria for insomnia disorder, and the Chinese Classification and Diagnostic Criteria of Mental Disorders, third edition (CCMD-3) (11) established by the Chinese Society of Psychiatry. For TCM diagnostic criteria, the standard is based on the “Guidelines for the Diagnosis and Efficacy Evaluation of TCM Syndromes” issued by the State Administration of Traditional Chinese Medicine in 2012. Additionally, participants were required to have a PSQI score of ≥ 6. Treatment duration ranged from 2 weeks (12–14) to 12 weeks (15). The therapeutic interventions were systematically categorized into experimental and control modalities. The experimental groups received standardized acupuncture protocols. The control groups received standard Western medicine regimens, primarily consisting of benzodiazepines and non-benzodiazepine hypnotics, specifically alprazolam, diazepam, estazolam, and zolpidem, which represent the most commonly prescribed medications for sleep disorders in clinical practice. The quantitative synthesis, as detailed in Table 2, incorporated methodological rigor through the inclusion of 25 high-quality studies that directly compared the therapeutic efficacy of acupuncture versus pharmacological interventions. This comparative analysis enabled a comprehensive evaluation of both therapeutic modalities within a standardized methodological framework.

**Table 2:** Literature specific information table of randomized controlled trials of acupuncture for insomnia patients.

Number	Author	Year	Sample size	Group	Sex (M/F)	Average age (years)	Intervening measure	Treatment (weeks)	Main outcomes	Results
1	Li et al.	2020	128	T C	20/44 21/43	51.00 ± 12.50 18.60 ± 1.33	Acupuncture Oxazepam	4	PSQI	No significant difference in PSQI scores (*p* > 0.05)
2	Zhang et al.	2020	40	T C	7/13 5/15	29.00 ± 21.00 36.00 ± 19.00	Acupuncture Estazolam	4	PSQI	Significant differences in PSQI scores (*p* < 0.05)
3	Wang	2014	80	T C	15/25 14/26	51.40 ± 2.30 53.10 ± 2.10	Acupuncture Estazolam	4	PSQI	Significant differences in PSQI scores (*p* < 0.05)
4	Zhang et al.	2015	119	T C	23/38 23/34	44.98 ± 11.67 43.18 ± 12.08	Acupuncture Alprazolam	4	PSQI	Significant differences in PSQI scores (*p* < 0.05)
5	Ji et al.	2015	70	T C	17/18 19/16	37.00 ± 11.00 36.00 ± 13.00	Acupuncture Trazodone	4	PSQI	Significant differences in PSQI scores (*p* < 0.05)
6	Yan et al.	2020	126	T C	30/35 29/32	45.99 ± 3.76 46.83 ± 3.49	Acupuncture Estazolam	4	PSQI	Significant differences in PSQI scores (*p* < 0.05)
7	Feng et al.	2020	80	T C	21/19 22/18	41.43 ± 4.68 41.95 ± 4.53	Acupuncture Alprazolam	4	PSQI	Significant differences in PSQI scores (*p* < 0.05)
8	Hua et al.	2016	90	T C	22/23 23/22	26.90 ± 4.30 28.90 ± 5.00	Acupuncture Triazolam	2	PSQI	Significant differences in PSQI scores (*p* < 0.05)
9	Pan et al.	2020	60	T C	13/17 14/16	42.03 ± 5.82 43.26 ± 5.27	Acupuncture Estazolam	4	PSQI ISI	Significant differences in PSQI scores (*p* < 0.05) Significant differences in ISI scores (*p* < 0.05)
10	Wu et al.	2023	90	T C	22/23 24/21	43.00 ± 8.00 43.00 ± 8.00	Acupuncture Estazolam	4	PSQI	Significant differences in PSQI scores (*p* < 0.05)
11	Zhao et al.	2023	68	T C	19/15 20/14	45.96 ± 6.73 46.20 ± 6.38	Acupuncture Estazolam	4	PSQI	Significant differences in PSQI scores (*p* < 0.05)
12	Dong et al.	2020	60	T C	15/15 14/16	63.60 ± 23.10 65.20 ± 4.00	Acupuncture Estazolam	4	PSQI	Significant differences in PSQI scores (*p* < 0.05)
13	Ti	2020	100	T C	24/26 22/28	49.92 ± 16.25 51.44 ± 16.28	Acupuncture Estazolam	4	PSQI	Significant differences in PSQI scores (*p* < 0.05)
14	Wang et al.	2017	90	T C	11/34 13/32	41.50 ± 4.60 39.10 ± 5.20	Acupuncture Mirtazapine	12	PSQI	Significant differences in PSQI scores (*p* < 0.05)
15	Xie	2018	83	T C	22/21 21/19	56.94 ± 9.83 58.15 ± 12.20	Acupuncture Estazolam	4	PSQI	Significant differences in PSQI scores (*p* < 0.05)
16	Jiang et al.	2022	84	T C	24/18 22/20	48.17 ± 6.64 47.63 ± 7.02	Acupuncture Right zopiclone	4	PSQI	Significant differences in PSQI scores (*p* < 0.05)
17	Wu et al.	2021	59	T C	12/18 10/19	41.00 ± 10.00 42.00 ± 10.00	Acupuncture Estazolam	4	PSQI	Significant differences in PSQI scores (*p* < 0.05)
18	Huang et al.	2024	62	T C	17/14 13/18	61.05 ± 7.63 61.85 ± 8.22	Acupuncture Estazolam	4	PSQI	Significant differences in PSQI scores (*p* < 0.05)
19	Liu	2017	70	T C	21/14 20/15	62.08 ± 7.82 63.02 ± 7.79	Acupuncture Estazolam	4	PSQI	Significant differences in PSQI scores (*p* < 0.05)
20	Zhang et al.	2021	60	T C	14/16 12/18	48.20 ± 6.74 47.33 ± 6.58	Acupuncture Estazolam	2	PSQI	Significant differences in PSQI scores (*p* < 0.05)
21	Hu et al.	2024	150	T C	31/44 34/41	52.21 ± 5.05 52.17 ± 5.24	Acupuncture Clonazepam	8	PSQI	Significant differences in PSQI scores (*p* < 0.05)
22	Wang et al.	2016	68	T C	9/25 11/23	53.00 ± 13.43 53.00 ± 11.37	Acupuncture Estazolam	4	PSQI	Significant differences in PSQI scores (*p* < 0.01)
23	Shao et al.	2017	112	T C	20/36 22/34	44.60 ± 13.50 45.80 ± 14.10	Acupuncture Zopiclone	4	PSQI	Significant differences in PSQI scores (*p* < 0.05)
24	Yin et al.	2017	60	T C	13/17 11/19	43.43 ± 13.52 46.80 ± 12.95	Acupuncture Estazolam	4	PSQI	Significant differences in PSQI scores (*p* < 0.05)
25	Pan et al.	2017	80	T C	23/17 24/16	55.50 ± 3.10 55.70 ± 3.20	Acupuncture Diazepam	2	PSQI	Significant differences in PSQI scores (*p* < 0.05)

T, Experimental group; C, Control group; PSQI, Pittsburgh sleep quality index; ISI, insomnia severity index.

### 3.3 Methodological quality

While the majority of acupuncture studies for insomnia disorder have demonstrated excellent performance in random sequence generation and allocation concealment, implementing blinding for participants and staff has been challenging due to the inherent nature of the treatment. Conversely, there was minimal potential for bias concerning outcome assessor blinding, data completeness, and reporting selectivity.

**Figure 2:**
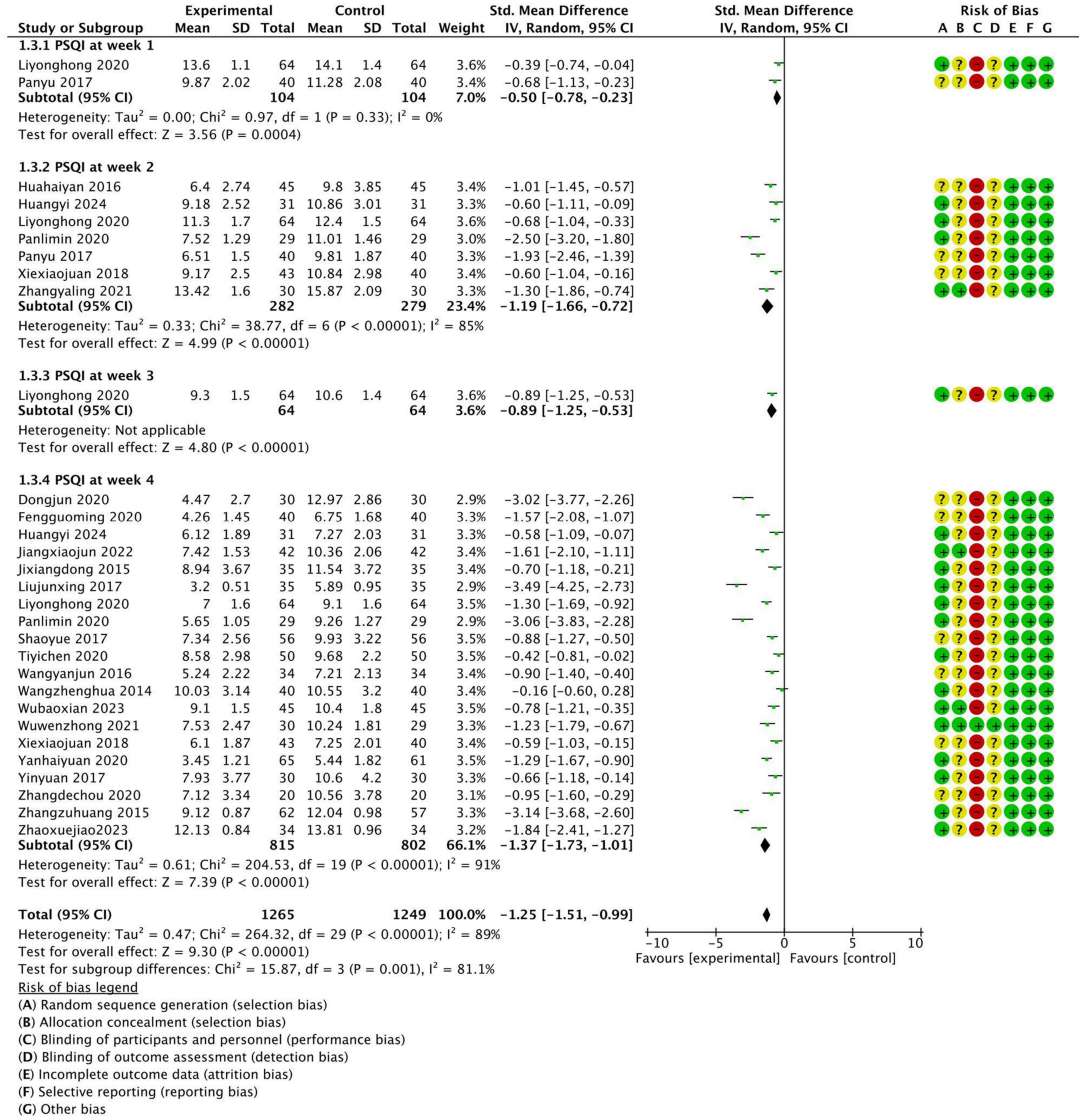
Weekly meta-analysis and bias assessment of acupuncture treatment for patients with insomnia according to the Pittsburgh Sleep Quality Index (PSQI).

The methodological quality of studies involving patients with insomnia disorder during weeks 1, 2, 3, and 4, as measured by PSQI scores, is summarized in Figure 2, based on the 23 articles ultimately included in the analysis. The results of the Cochrane risk of bias (ROB) analysis were largely consistent. Eighteen studies exhibited low ROB in terms of random sequence generation and allocation concealment. However, all studies showed high ROB for participant and staff blinding. Additionally, all studies maintained low ROB regarding incomplete outcome data and blinded outcome assessment.

### 3.4 Quality assessment of the included studies

**Figure 3:**
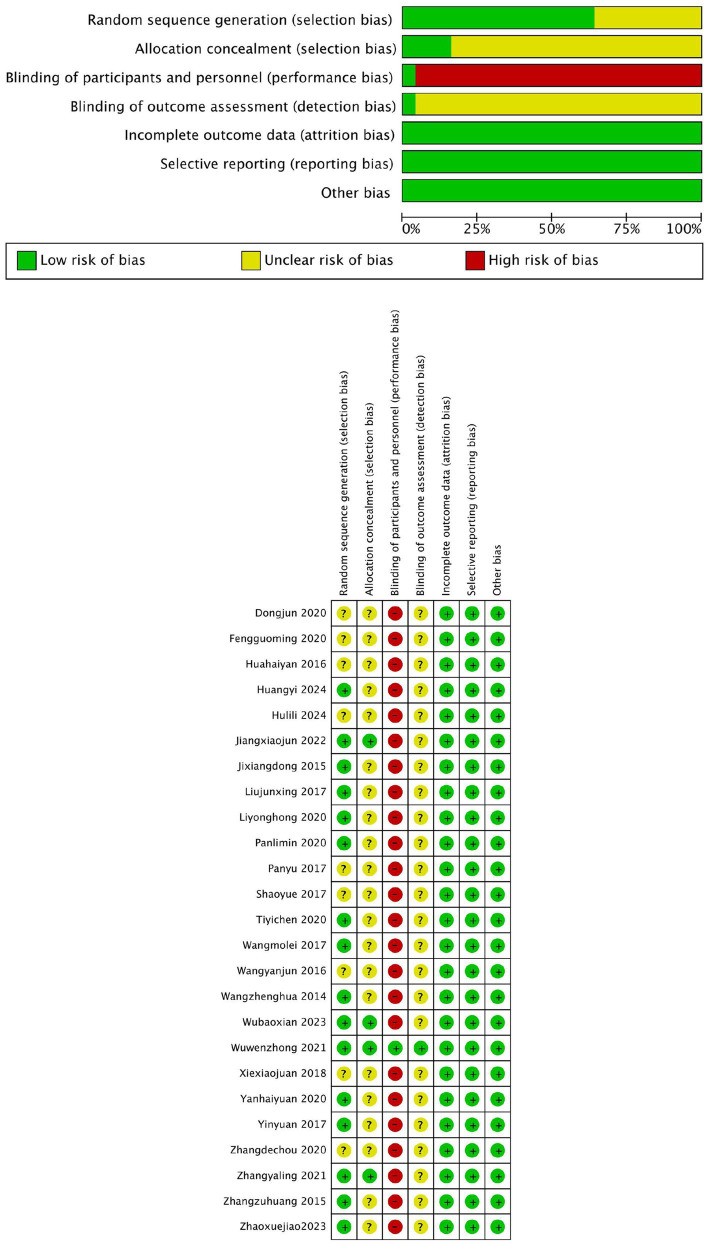
Bias in inclusion of literature.

This study assessed the quality of 25 randomized controlled trials using the Cochrane Risk of Bias tool. Regarding random sequence generation, 11 studies (15–25) employed the random number table method, 3 studies (26–28) used sealed envelope randomization, and 2 studies (13, 29) generated random numbers via computer. The remaining 9 studies (12, 14, 30–36) only mentioned the term “randomization.” For allocation concealment, 4 studies (13, 26–28) used sealed opaque envelopes, while the remaining 21 studies did not clearly report allocation concealment methods. Regarding blinding of researchers and participants, due to the limitations of the interventions, not all studies adopted double-blinding. Only one study (28) blinded the assessors, while the other 24 studies had a high risk of performance bias. For blinding of outcome assessors, one study (28) ensured that outcome assessors were blinded, whereas the remaining 24 studies did not explicitly report blinding of outcome assessors. In terms of incomplete outcome data, selective reporting, and other biases, all studies reported complete data, with no evidence of selective reporting or other biases. The overall risk of bias assessment indicated that the primary risks were concentrated in the lack of allocation concealment and blinding implementation, while random sequence generation and data completeness performed relatively well. The risk of bias was visualized using Review Manager 5.4 software, as shown in Figure 3.

### 3.5 Meta-analysis

#### 3.5.1 Clinical trial efficacy and result analysis

**Figure 4:**
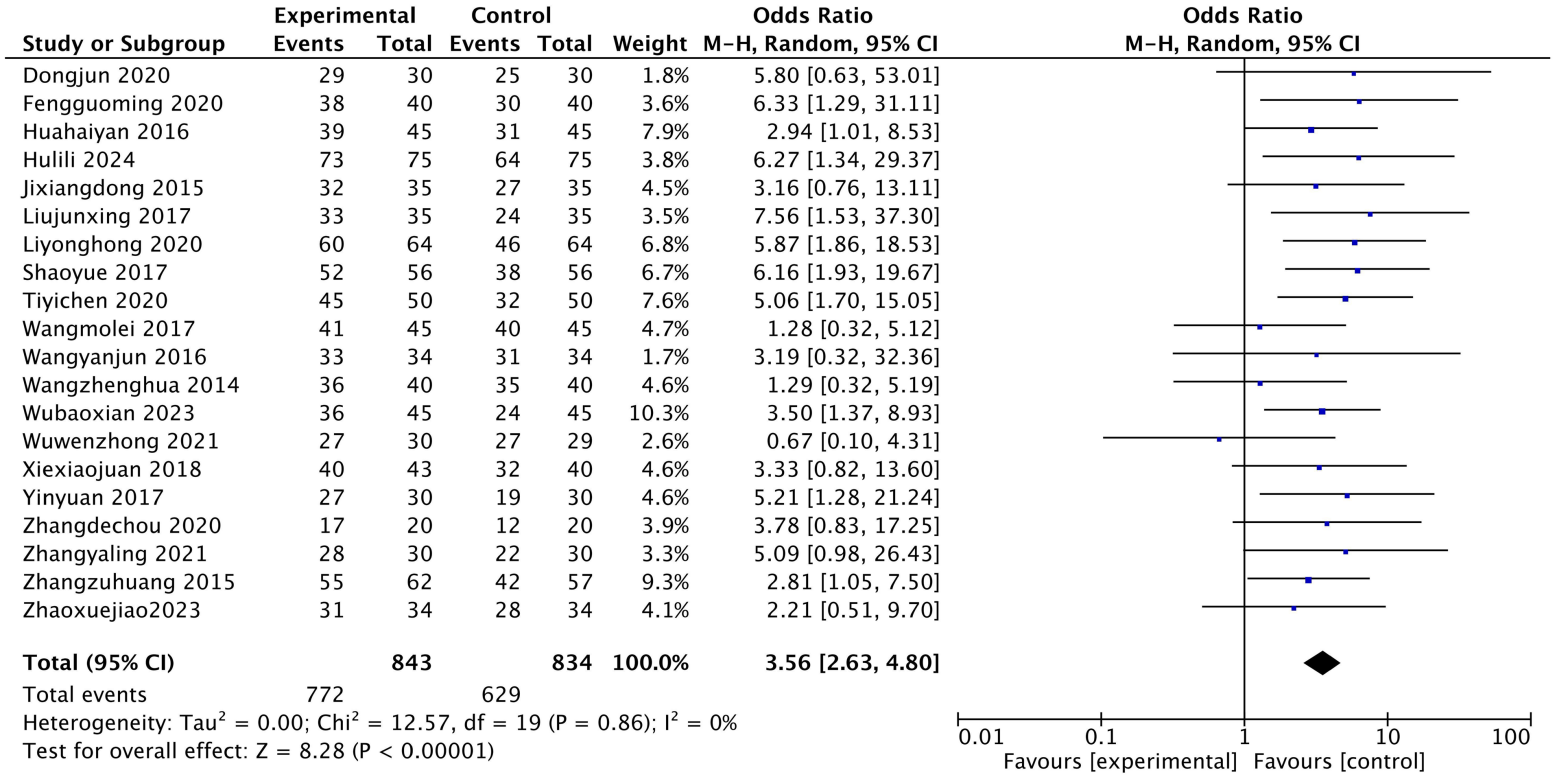
Efficient Meta Forest Map.

The efficacy observation index included in the literature was the effective rate of treatment. A dichotomous classification of count data was used to test the efficacy rates described in the literature. In this study, the effective rate of treatment was determined as the sum of the clinical cure rate, effective rate, and substantial effective rate recorded in the literature, and the ineffective rate was defined as the zero point of effective treatment. The results of 20 studies were tested for heterogeneity. The results showed that there was homogeneity among the studies, I^2^ = 0; The random effect model was used for statistical analysis, and the pooled OR value was 3.56. The rhomboid was located on the right side of the midline, 95% CI was [2.63, 4.80]; Z test: acupuncture group vs. control group Z = 8.28, *p* < 0.00001. As shown in Figure 4, the therapeutic effect of the acupuncture group was statistically different from that of the traditional western medicine treatment.

#### 3.5.2 Assessment of acupuncture’s effect on PSQI scale scores

**Figure 5:**
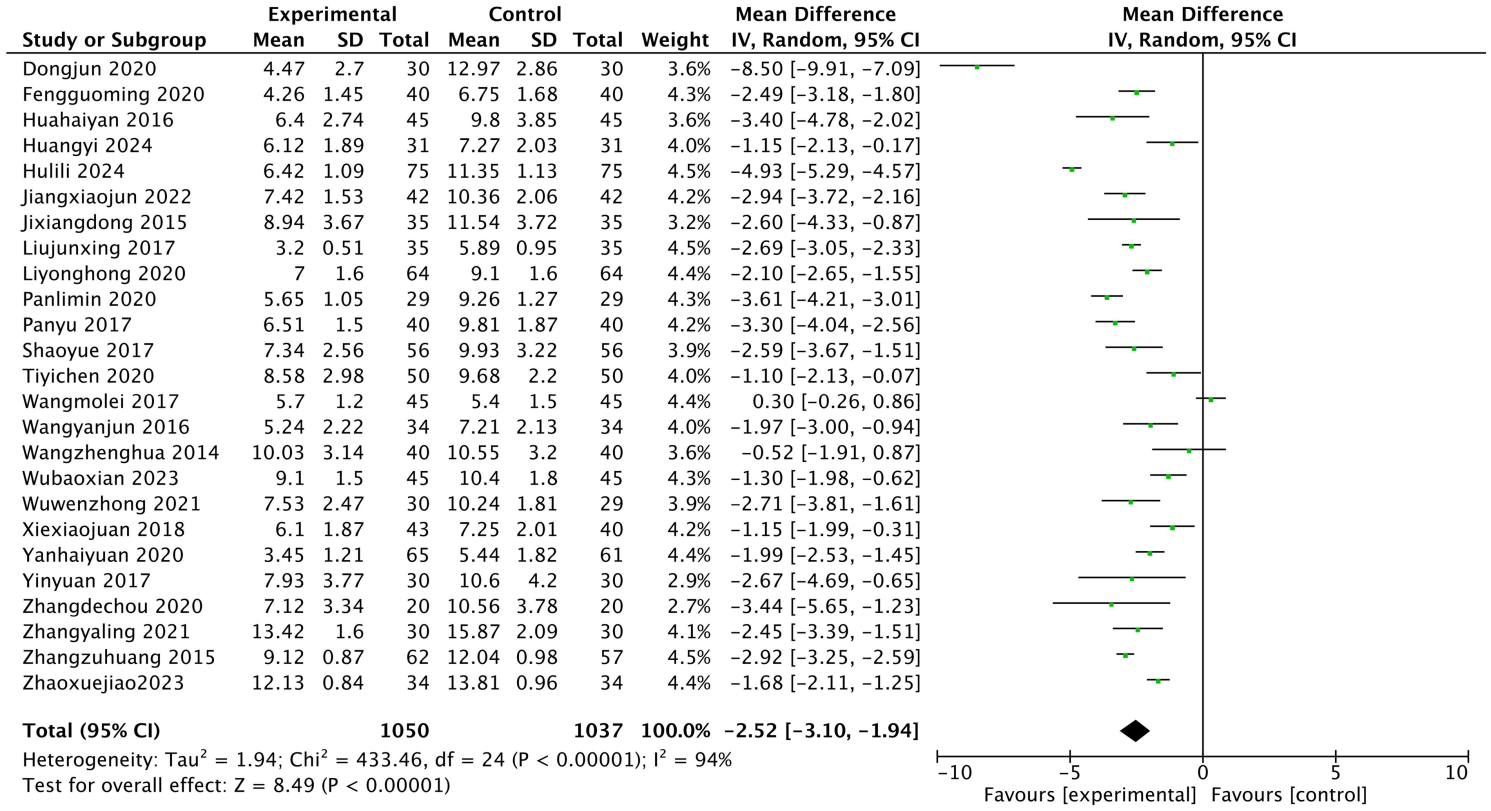
Pittsburgh Sleep Quality Index (PSQI) score meta-analysis forest plot.

The heterogeneity test of the results of 25 studies showed that there was a high degree of heterogeneity among the studies (I^2^ = 94%). The random effect model was used for statistical analysis, and the diamond was on the left side of the midline. The pooled MD value was −2.52, and the 95% CI was [−3.10, −1.94]. Z test showed that the difference was statistically significant (Z = 8.49, *p* < 0.00001). After treatment, PSQI in the experimental acupuncture group was lower than that in the western medication control group, as shown in Figure 5 (PSQI score meta-analysis forest plot).

#### 3.5.3 Funnel plot and result analysis

**Figure 6:**
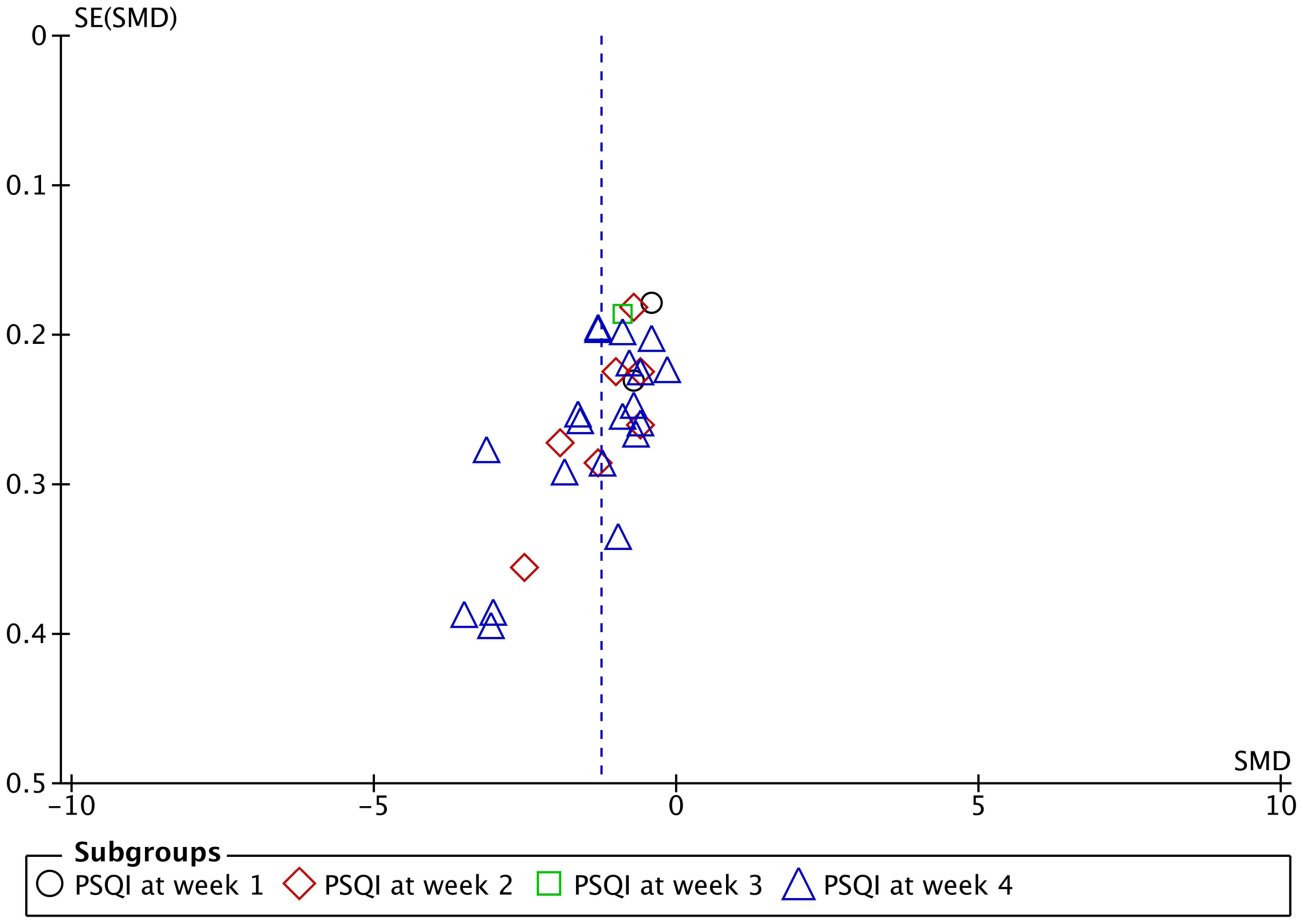
The funnel plot representing the publication bias analyses Pittsburgh Sleep Quality Index (PSQI) for acupuncture treatment of 1, 2, 3, and 4 weeks.

In terms of publication bias, funnel plot analysis was performed on the studies included in the quantitative analysis according to PSQI scores, which were mainly concentrated in the middle and relatively symmetrical, suggesting that there was no large publication bias. All 25 studies used the overall response rate as a measure of efficacy after treatment, as shown in Figure 6.

#### 3.5.4 Comparison of time-stratified efficacy of PSQI scores

A comprehensive meta-analysis (Figure 2), based on PSQI scores across different treatment weeks confirmed that acupuncture is a significant treatment method for insomnia disorder (SMD: −1.25; 95% CI: −1.51 to −0.99; *p* < 0.00001; I^2^ = 89%; *N* = 2,514). Among the 23 included studies, the treatment durations were 1 week, 2 weeks, 3 weeks, and 4 weeks, respectively. Two studies (14, 16) did not find a significant effect of acupuncture in the first week (SMD: −0.50; 95% CI: −0.78 to −0.23; *p* = 0.0004; I^2^ = 0; *N* = 208). Seven studies (12–14, 16, 23, 29, 33) demonstrated a significant effect of acupuncture on insomnia disorder patients by the second week (SMD: −1.19; 95% CI: −1.66 to −0.72; *p* < 0.00001; I^2^ = 85%; *N* = 561). One study (16) highlighted a pronounced advantage by the third week (SMD: −0.89; 95% CI: −1.25 to −0.53; *p* < 0.00001; *N* = 128). Finally, at 4 weeks, 20 studies (16–33, 35, 36) emphasized the significant benefits of acupuncture (SMD: −1.37; 95% CI: −1.73 to −1.01; *p* < 0.00001; I^2^ = 91%; *N* = 1,617).

### 3.6 Acupuncture points

A total of 52 acupuncture points were utilized across the 25 studies, with each point applied in various combinations. As shown in Table 3 the most frequently used point combinations involved HT7, SP6, and GV20, often paired with PC6 or EX-HN1 to address different insomnia disorder subtypes. These points were categorized based on their frequency of use: the most commonly used points (>75% of studies) included HT7, SP6, GV20, EX-HN1, and PC6; those used in 50–75% of studies (commonly used) were GB20, BL15, LR3, KI1, Extra, EX-HN3, BL62, KI6, and KI3; and points used in 25–50% of studies (often used) comprised GV24, PC4, BL1, BL20, BL13, BL23, LI4, BL18, LR14, Emotional Area (Forehead Region), ST36, GV16, GB15, GB8, GB41, GB43, BL7, BL8, BL9, GB17, GB18, BL17, HT6, HT5, CV4, CV6, GV23, GV22, GV21, GV19, Jiaji Points (T5, T9, T10), CV12, BL10, SP9, and TE5. Additionally, less frequently applied points such as Ashi Points (Head Region) were used in 10–25% of studies. The number of acupuncture points administered per insomnia disorder patient varied across trials, reflecting the adaptability of protocols to individual symptom patterns. From the perspective of traditional Chinese medicine theory, several commonly used acupoints, such as HT7, as the original point of the Heart Meridian, is mainly used to calm the mind and soothe the spirit, targeting “restlessness of the heart and spirit.” SP6, as the convergence point of the liver, spleen and kidney meridians, mainly regulates qi and blood, targeting “qi and blood imbalance.” GV20, as a key point on the Governor Vessel, is mainly used to calm the mind and soothe the spirit, targeting the condition of “excessive Yang disturbing the spirit.” The combination of the three acupoints can not only connect the heart and kidneys to treat “insufficiency of water and fire,” but also soothe the liver and strengthen the spleen to relieve “emotional distress,” and even elevate the clear and reduce the turbid to calm “hyperactivity of liver Yang.” This acupoint selection plan conforms to the holistic view of traditional Chinese medicine of “meridians-zang-fu organs-mental state,” and achieves the therapeutic goal of “regulating the spirit and promoting sleep.”

**Table 3:** Acupuncture Points Selected for the Treatment of Insomnia.

	Frequency of use	Acupuncture points
Acupuncture	Most commonly used (in >75% of studies)	HT7, SP6, GV20, EX-HN1, PC6
	Commonly used (in 50–75% of studies)	GB20, BL15, LR3, KI1, Extra, EX-HN3, BL62, KI6, KI3
	Often used (in 25–50% of studies)	GV24, PC4, BL1, BL20, BL13, BL23, LI4, BL18, LR14, Emotional Area (Forehead Region), ST36, GV16, GB15, GB8, GB41, GB43, BL7, BL8, BL9, GB17, GB18, BL17, HT6, HT5, CV4, CV6, GV23, GV22, GV21, GV19, Jiaji Points (T5, T9, T10), CV12, BL10, SP9, TE5
	Sometimes used (in 10–25% of studies)	Ashi Points (Head Region)

## 4 Discussion

In recent years, insomnia disorder has become one of the most serious health issues among middle-aged and elderly people, with its incidence significantly rising due to increasing life stress (37). Insufficient sleep duration and poor sleep quality not only severely affect patients’ daily lives and work efficiency but may also lead to a series of psychological and physiological problems. Consistent with the DSM-V (9), insomnia is now diagnosed as Insomnia Disorder, which may occur independently or comorbidly with other medical or psychiatric conditions (e.g., anxiety, depression, chronic pain). Its main symptoms include difficulty falling asleep, poor sleep quality, short sleep duration, decreased attention, memory loss, daytime sleepiness, fatigue, and even life-threatening signs such as palpitations and chest tightness. In TCM theory, insomnia disorder is considered to fall under the category of “Bù Mèi” (38), and its pathogenesis is closely related to the imbalance of yin and yang. TCM emphasizes that the alternation of yin and yang determines the cycle of sleep and wakefulness and believes that sleep is closely related to mental state. The brain, as the residence of “original qi,” should be treated by regulating the balance of yin and yang and mental state.

Currently, although drug treatment is a common approach, it has limitations such as slow onset and numerous adverse reactions. While CBT-I is recognized as the first-line non-pharmacological treatment, our findings demonstrate that acupuncture serves as a clinically significant alternative to sedative-hypnotic medications.

In contrast, acupuncture therapy stands out for its significant clinical efficacy and high safety. The results of this study show that the improvement in PSQI scores in the acupuncture treatment group was significantly better than that in the control group (*p* < 0.05), with no significant adverse reactions observed. In contrast, the control group using conventional sedative-hypnotic medications treatment, although it improved sleep quality through sedative effects, had an adverse reaction rate as high as 19.35%, with common side effects including dry mouth, dizziness, drowsiness, and fatigue (23). In summary, acupuncture therapy demonstrates significant advantages in improving insomnia disorder symptoms and enhancing sleep quality, while also offering high safety, providing a safer and more effective treatment option for insomnia disorder patients.

### 4.1 Efficacy and safety of acupuncture for insomnia disorder

After a systematic review of 3,100 articles, only 25 studies (including a total of 2087 patients diagnosed with insomnia disorder) met the stringent inclusion and exclusion criteria. Without restricting the duration or severity of the condition, we selected 23 of these studies for a comprehensive quantitative meta-analysis, using the PSQI score and treatment efficacy rate as the primary evaluation indicators. The meta-analysis results showed that, across all 25 RCTs, patients receiving acupuncture treatment demonstrated significantly greater symptom improvement and lower incidence of adverse reactions and side effects compared to those receiving conventional pharmacological treatment. The significant improvement in PSQI score provides strong evidence for the effectiveness of acupuncture in treating sleep quality in patients with insomnia disorder, especially considering that PSQI is an effective tool for assessing subjective sleep quality in patients with sleep disorders, including insomnia disorder.

The significant therapeutic effect of acupuncture for insomnia disorder is supported by a pooled meta-analysis that included 23 clinical trials using PSQI scores as an evaluation metric (RR: −1.25; 95% CI: −1.51 to −0.99; *p* < 0.00001; I^2^ = 89%; *N* = 2,514). Clinical practice has demonstrated that acupuncture treatment for insomnia disorder yields significant clinical outcomes. Furthermore, multiple clinical studies in recent years have further confirmed that the safety and efficacy of acupuncture for insomnia disorder have significantly improved, providing more reliable evidence for its application in clinical settings.

Included in this review are 25 studies on acupuncture for the treatment of insomnia disorder. The majority of these studies employed standardized acupuncture protocols, although there were variations in frequency and treatment modalities between the intervention and control groups. Our analysis indicates that acupuncture demonstrates efficacy comparable to pharmacological treatments within the initial 1–3 weeks. However, by the fourth week, the therapeutic effect of acupuncture surpasses that of medications. This finding underscores the potential of acupuncture as a standalone therapy within depression treatment programs. For individualized patient care, practitioners may consider acupuncture as a complementary approach to pharmacological treatment, particularly for patients with chronic conditions, poor response to medications, significant adverse effects, or those seeking non-pharmacological interventions. Acupuncture also serves as an alternative treatment option for patients who are hesitant about medications. The findings suggest that a treatment duration of at least 4 weeks is necessary and can serve as a reference point in clinical practice, assisting clinicians in refining treatment plans and evaluating efficacy. While acupuncture has demonstrated significant efficacy in treating insomnia disorder, its exact mechanisms of action warrant further investigation. Additionally, future research should continue to explore the effects of various acupuncture modalities, techniques, treatment frequencies, and durations on therapeutic outcomes. In future RCTs, more comparisons between acupuncture and other non-pharmacological treatments, such as massage, moxibustion, and psychotherapy, are warranted to further evaluate the benefits of acupuncture for insomnia disorder.

### 4.2 Mechanism of acupuncture for insomnia disorder

#### 4.2.1 Mechanism of acupuncture in treating insomnia disorder from the perspective of traditional Chinese medicine (TCM)

In TCM, insomnia disorder (“Bù Mèi”) is primarily associated with heart dysfunction, often linked to imbalances in the liver, spleen, and kidneys (39). The most common pattern, heart-spleen deficiency (40, 41), arises from emotional stress, fatigue, or disturbed heart spirit, leading to yin-yang imbalance and disrupted qi-blood circulation (42). Acupuncture treats insomnia disorder by harmonizing yin-yang, unblocking meridians, and regulating zang-fu organs. Through acupoint stimulation, it reduces excess yang, supplements deficient yin (43), and restores qi-blood flow in the heart, liver, spleen, and kidneys, thereby calming the mind. Specific approaches include strengthening the spleen, nourishing the heart, soothing the liver, and tonifying the kidneys to resolve mental restlessness. Additionally, regulating the Governor Vessel (Du Mai) further stabilizes the heart spirit and promotes sleep (28).

#### 4.2.2 Mechanism of acupuncture in treating insomnia disorder from the perspective of western medicine

Modern research suggests that acupuncture treats insomnia disorder by modulating central neurotransmitters, inflammatory factors, and the neuroendocrine system. By stimulating specific acupoints, acupuncture regulates the neuro-humoral system, promoting neurotransmitter release and improving immune function (44–47). It increases 5-HT (serotonin) while reducing NE (norepinephrine), thus stabilizing the sleep–wake cycle (18, 48). Additionally, acupuncture lowers pro-inflammatory cytokines (IL-6, TNF-*α*), reducing their disruption of sleep (12, 47). Acupuncture also balances GABA (inhibitory) and Glu (excitatory) neurotransmitters, enhancing sleep quality (32, 48–50). It modulates the hypothalamic–pituitary–adrenal (HPA) axis, lowering cortisol (CORT) levels to alleviate stress-related insomnia disorder (28, 47). The latest research also found that acupuncture can up-regulate BDNF, promote neurological function and improve sleep regulation (48, 51–53). Through these multitarget mechanisms, acupuncture improves sleep architecture by regulating the neuro-endocrine-immune network. Furthermore, studies have shown that acupuncture can significantly reduce the PSQI score (with an average reduction of 2.52 points). This improvement is not only statistically significant but also clinically equivalent to an increase of more than 45 min in sleep time, meeting the clinical standard for improved sleep quality (8).

### 4.3 Acupuncture treatment regimen

There is significant variability in the selection of acupoints across the included studies. A total of 25 articles were reviewed, identifying 52 acupoints. Among these, HT7, SP6, GV20, EX-HN1, and PC6 being the most frequently used (application rates >75%), followed by GB20, BL15, LR3, KI1, and others (>50%). Additional points like GV24, ST36, LI4, and Ashi points were also considered.

The variability in acupoint selection across studies underscores a key principle of TCM: treatment must be tailored to the individual’s condition, reflecting the holistic and adaptive nature of acupuncture. Despite differing point combinations, the frequent use of HT7, SP6, GV20, and other major acupoints suggests a consensus on their therapeutic importance, particularly for conditions involving the upper body and head—regions requiring precise technique due to minimal tissue protection. The shared emphasis on prolonged treatment duration (20–30 min over ≥ 4 weeks) further supports that consistency in stimulation, rather than rigid point selection, is critical for efficacy. Thus, while methods vary, the findings collectively affirm acupuncture’s patient-centered approach, where flexibility in application aligns with TCM’s overarching goal of restoring systemic balance.

### 4.4 Comparison between acupuncture and western medicine in treating insomnia disorder

Acupuncture demonstrates significant advantages in efficacy, safety, and personalized treatment for insomnia disorder. Compared to sedative-hypnotic medications, acupuncture not only significantly improves sleep quality, prolongs sleep duration, and reduces nighttime awakenings but also effectively alleviates accompanying anxiety, depression, and other emotional disorders, with no risk of drug dependency or significant side effects, making it suitable for long-term use. While sedative-hypnotic medications provides rapid relief of insomnia disorder symptoms in the short term, long-term use may lead to drug dependency and tolerance, accompanied by adverse reactions such as dizziness, fatigue, and decreased attention, and may even exacerbate emotional issues. Additionally, acupuncture emphasizes syndrome differentiation and treatment, tailoring therapies to the patient’s specific symptoms, constitution, and etiology, with flexible adjustments to acupoints and techniques. Studies have also shown that acupuncture significantly outperforms conventional sedative-hypnotic medications in improving sleep structure, sleep quality, and episodic memory function in patients with chronic insomnia disorder, particularly in regulating neurotransmitter balance, enhancing brain function, and alleviating chronic stress. Therefore, as a safe, effective, sustainable, and non-dependent treatment modality, acupuncture holds significant clinical value in insomnia disorder treatment, especially for patients concerned about medication or those suffering from long-term insomnia disorder.

### 4.5 Clinical application prospects of acupuncture in treating insomnia disorder

Acupuncture presents a highly effective and safe alternative for insomnia disorder treatment, demonstrating unique advantages through its ability to regulate neuroendocrine functions to improve sleep quality, prolong duration, and reduce nighttime awakenings while alleviating associated symptoms like anxiety and depression. Unlike pharmacological interventions, it eliminates risks of drug dependency and minimizes side effects, particularly suitable for long-term management. Its personalized approach through syndrome differentiation allows tailored acupoint selection and techniques to optimize outcomes, while compatibility with other therapies like herbal medicine and psychotherapy enables comprehensive treatment strategies. Supported by growing clinical evidence and recognized for its cost-effectiveness and cultural acceptance, acupuncture is increasingly incorporated into healthcare systems worldwide, offering a sustainable therapeutic option for insomnia disorder patients.

## 5 Limitations

There are several limitations to this study that warrant attention. Firstly, TCM treatment regimens are highly individualized and challenging to standardize, which complicates the comparability of results across studies. This heterogeneity in control group settings, including variations in sex distribution, insomnia disorder severity, and disease duration, further exacerbates this challenge. Secondly, differences in acupoint selection, manipulation techniques, and stimulation intensity within clinical settings, even among evidence-based treatments within the same study, may contribute to inconsistent outcomes. Thirdly, our focus on RCTs conducted within the past decade reflects an emphasis on timeliness and relevance; however, this approach may introduce potential biases. A critical limitation is the lack of long-term follow-up assessments in the included studies. While the meta-analysis demonstrated significant short-term improvements in PSQI scores, the sustainment of these results over time was not evaluated. Without follow-up data, it cannot be assumed that the therapeutic benefits of acupuncture persist beyond the immediate treatment period. This gap underscores the need for future research to incorporate extended observation periods to assess the durability of acupuncture’s effects on insomnia disorder. Additionally, areas such as acupoint selection, specific acupuncture types, methodology, and variability in double-blind restrictions require further refinement to enhance the robustness of future conclusions. Fourthly, few articles reported the incidence of adverse reactions, and the stability of the evidence network was suboptimal, potentially affecting result accuracy. Clinical staff should exercise caution accordingly. Fifthly, PSQI was solely adopted as the primary outcome measure, while objective indicators (such as polysomnography and actigraphy) and the Insomnia Severity Index (ISI) - a widely used assessment tool for insomnia disorder severity - were not included. PSQI relies on patient self-report rather than objective sleep measures. While the PSQI is a validated and widely used instrument, self-reported assessments may be subject to recall bias and placebo effects. Future studies could strengthen these findings by incorporate both subjective and objective measures to provide a comprehensive evaluation of treatment efficacy. Lastly, this study is limited by a small sample size, short observation period, lack of long-term follow-up data, non-standardized acupuncture treatment plans, subjective acupoint selection procedures, and an efficacy evaluation system that requires further refinement. These factors may compromise the accuracy and reliability of the findings.

## 6 Conclusion

This study systematically compared the therapeutic efficacy between conventional Western medicine and traditional Chinese acupuncture in treating insomnia disorder. A meta-analysis of 25 randomized controlled trials demonstrated that after 3–4 weeks of acupuncture treatment, patients showed significantly greater improvement in sleep quality (mean PSQI score reduction: 2.52 points, 95% CI: −3.10 to −1.94, *p* < 0.00001) and insomnia disorder severity compared to pharmacotherapy, with the treatment effect size exceeding the minimal clinically important difference for PSQI. Modern medical research confirms that acupuncture exerts its therapeutic effects by regulating sleep-related neurotransmitters and hormone levels, while avoiding the common dependency risks and cognitive side effects associated with drug therapy, demonstrating its unique safety advantages. Although the current study has limitations including relatively small sample sizes and short follow-up periods (heterogeneity I^2^ = 94%), the favorable efficacy and safety profile of acupuncture suggests it may be particularly valuable for patients seeking non-pharmacological alternatives or those concerned about medication side effects. Future research should focus on conducting multicenter, large-sample clinical trials, establishing standardized treatment protocols, and incorporating objective sleep assessment measures to further validate the clinical value of acupuncture and provide safer, more effective treatment options for insomnia disorder patients.

## Data Availability

The original contributions presented in the study are included in the article/supplementary material, further inquiries can be directed to the corresponding author.
