# Correction: Clinical efficacy and safety of acupuncture versus Western medicine for insomnia: a systematic review and meta-analysis

**DOI:** 10.3389/fneur.2025.1750057

**Published:** 2025-12-09

**Authors:** Jun Ma, Meng Peng, Xue-Jiao Xu

**Affiliations:** Heilongjiang University of Chinese Medicine, Harbin, Heilongjiang, China

**Keywords:** insomnia, acupuncture, Pittsburgh Sleep Quality Index, meta-analysis, systematic review

In the published article, errors were present in Figures and their corresponding in-text citations. The corrections are detailed below.

The reference “1” was erroneously written as “Xin ZZ, Peng Z, Qing HL. Guidelines for the diagnosis and treatment of adult insomnia in China. Chin. J Neurol. (2012) 7:534–40”. It should be “Zhao ZX, Zhang P, Huang LQ. Guidelines for the diagnosis and treatment of adult insomnia in China. *Chin J Neurol*. (2012) 7:534–40”.

The reference “7” was erroneously written as “Qi S. Acupuncture at Baihui and the Four Shencong Points for Treating Depression-Related Insomnia in 56 Cases. Capital Med. (2007) 18:48–9”. It should be “Song Q. Acupuncture at Baihui and the Four Shencong points for treating depression-related insomnia in 56 cases. *Capital Med*. (2007) 18:48–9”.

The reference “28” was erroneously written as “Wu WZ, Zheng SY, Liu CY, Qin S, Wang XQ, Li Hu Jin, et al. Effect of Tongdu Tiaoshen acupuncture on serum GABA and CORT levels in patients with chronic insomnia. Chinese Acupuncture & Moxibustion. (2021) 41:721–4. doi: 10.13703/j.0255-2930.20200704-k0001”. It should be “Wu WZ, Zheng SY, Liu CY, Qin S, Wang XQ, Hu JL, et al. Effect of *Tongdu Tiaoshen* acupuncture on serum GABA and CORT levels in patients with chronic insomnia. *Chin Acupunct Moxibustion*. (2021) 41:721–4. doi: 10.13703/j.0255-2930.20200704-k0001”.

The reference “32” was erroneously written as “Dong J, Ding JY, Zhang YY, Xiang SY, Huashun CUI. Clinical Observation of Acupuncture on the Treatment of Insomnia with Syndrome of Internal Disturbance of Phlegm-heat. World. J Sleep Med. (2020) 7:606–8. doi: 10.3969/j.issn.2095-7130.2020.04.019”. It should be “Dong J, Ding JY, Zhang YY, Xiang SY, Cui HS. Clinical observation of acupuncture on the treatment of insomnia with syndrome of internal disturbance of phlegm-heat. *World J Sleep Med*. (2020) 7:606–8. doi: 10.3969/j.issn.2095-7130.2020.04.019”.

The reference for “41” was erroneously written as “Tong X. The correlation research of TCM Ssyndrome and constitution oninsomnia and constitution. Harbin: Heilongjiang University of Chinese Medicine (2013)”. It should be “Tong X. *The Correlation Research of TCM Syndrome and Constitution on Insomnia* [Dissertion]. Heilongjiang University of Chinese Medicine, Harbin (2013)”.

The reference for “53” was erroneously written as “Wang H, Kun L, Hua ZY. Effect of Suanzaoren decoction on Bcl-2 and brain-derived neurotrophic factor mRNA in dorsal raphe nucleus of midbrain in rats with insomnia. Shi Zhen National Med Chinese Med. (2013) 24:1898–900. doi: 10.3969/j.issn.1008-0805.2013.08.040”. It should be “Wang H, Luo K, Zhao YH. Effect of Suanzaoren decoction on Bcl-2 and brain-derived neurotrophic factor mRNA in dorsal raphe nucleus of midbrain in rats with insomnia. *Shi Zhen Natl Med Chin Med*. (2013) 24:1898–900. doi: 10.3969/j.issn.1008-0805.2013.08.040”.

There were some formatting errors in Figure 1 as published. The corrected Figure 1 appears below.

**Figure 1:**
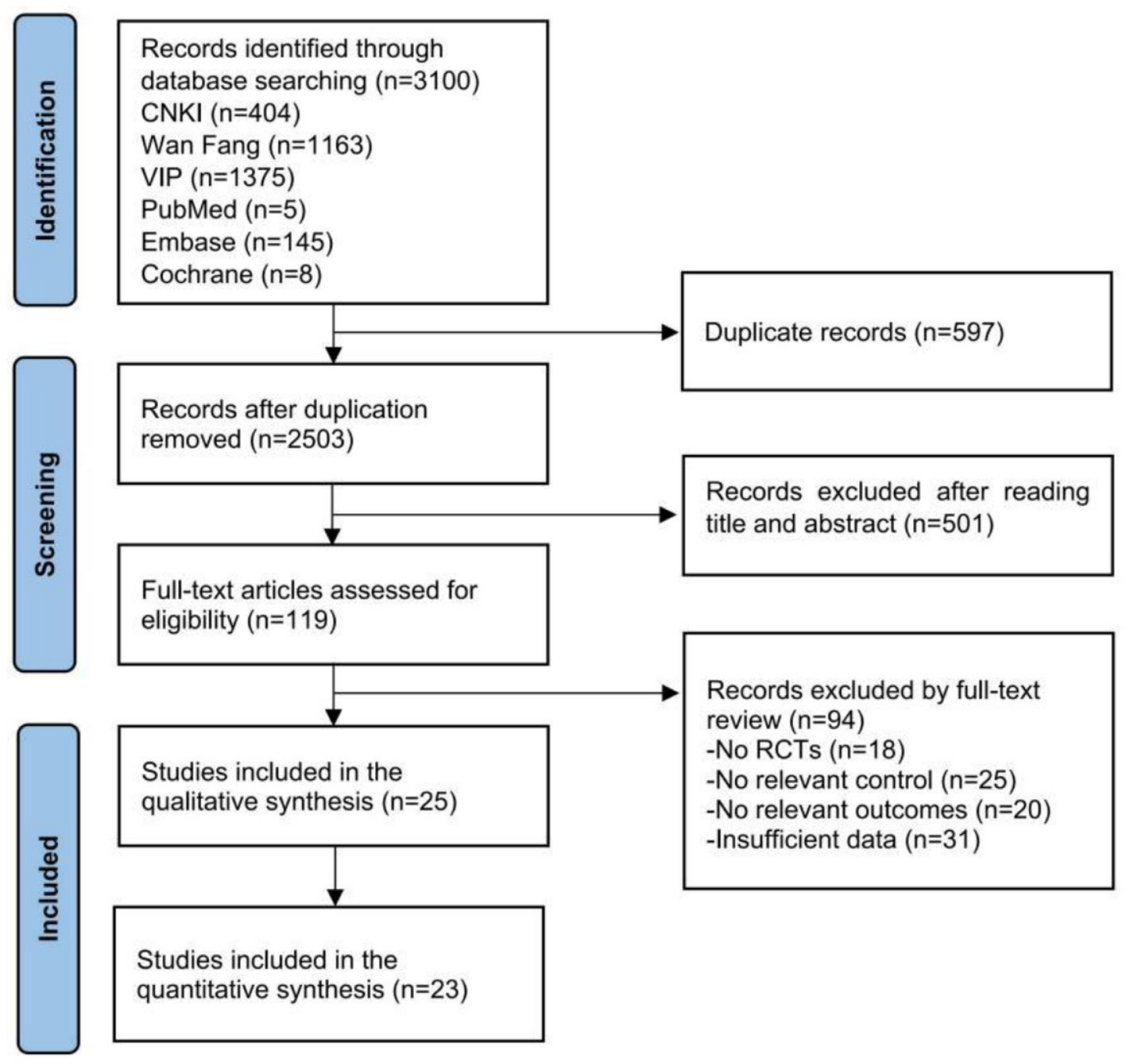
Flow chart of the review process.

There was an error in the captions of Figures 2–6 as published. The order and correspondence between figure numbers and their captions were incorrect. The corrected captions of Figures 2–6 appear below.

Figure 2. Weekly meta-analysis and bias assessment of acupuncture treatment for patients with insomnia according to the Pittsburgh Sleep Quality Index (PSQI).

Figure 3. Bias in inclusion of literature.

Figure 4. Efficient meta forest map.

Figure 5. Pittsburgh Sleep Quality Index (PSQI) score meta-analysis forest plot.

Figure 6. The funnel plot representing the publication bias analyses Pittsburgh Sleep Quality Index (PSQI) for acupuncture treatment of 1, 2, 3, and 4 weeks.

There were errors in the in-text citations of figures. Section 3.5.3 incorrectly cited Figure 2, while Section 3.5.4 correspondingly cited Figure 6 in error.

A correction has been made to Sections 3.5.3 and 3.5.4:

The correct correspondence is as follows:

## 3.5.3 Funnel plot and result analysis

In terms of publication bias, funnel plot analysis was performed on the studies included in the quantitative analysis according to PSQI scores, which were mainly concentrated in the middle and relatively symmetrical, suggesting that there was no large publication bias. All 25 studies used the overall response rate as a measure of efficacy after treatment, as shown in Figure 6.

The section 3.5.4 requires an update due to an error in figure citation; therefore, only the first sentence is listed here, while the content following the first sentence in this section remains unchanged from the original manuscript.

## 3.5.4 Comparison of time-stratified efficacy of PSQI scores

A comprehensive meta-analysis (Figure 2), based on PSQI scores across different treatment weeks confirmed that acupuncture is a significant treatment method for insomnia disorder (SMD: −1.25; 95% CI: −1.51 to −0.99; *p* < 0.00001; *I*^2^ = 89%; *N* = 2,514).

The original version of this article has been updated.

